# Caregivers’ understanding of dementia predicts patients’ comfort at death: a prospective observational study

**DOI:** 10.1186/1741-7015-11-105

**Published:** 2013-04-11

**Authors:** Jenny T van der Steen, Bregje D Onwuteaka-Philipsen, Dirk L Knol, Miel W Ribbe, Luc Deliens

**Affiliations:** 1 Department of General Practice & Elderly Care Medicine, , EMGO Institute for Health and Care Research, VU University Medical Center, Van der Boechorststraat 7, Amsterdam, 1081BT, The Netherlands; 2 Department of Public and Occupational Health, VU University Medical Center, EMGO Institute for Health and Care Research, Van der Boechorststraat 7, Amsterdam, 1081BT, The Netherlands; 3 VU University Medical Center, Department of Epidemiology and Biostatistics, VU University Medical Center, De Boelelaan 1118, Amsterdam, 1081HZ, The Netherlands; 4 Ghent University and Vrije Universiteit Brussels, End-of-Life Care Research Group, Laarbeeklaan 103, Brussels, 1090, Belgium

**Keywords:** Palliative care, End of life, Dementia

## Abstract

**Background:**

Patients with dementia frequently do not receive adequate palliative care which may relate to poor understanding of the natural course of dementia. We hypothesized that understanding that dementia is a progressive and terminal disease is fundamental to a focus on comfort in dementia, and examined how family and professional caregivers’ understanding of the nature of the disease was associated with patients’ comfort during the dying process.

**Methods:**

We enrolled 372 nursing home patients from 28 facilities in The Netherlands in a prospective observational study (2007 to 2010). We studied both the families and the physicians (73) of 161 patients. Understanding referred to families’ comprehension of complications, prognosis, having been counseled on these, and perception of dementia as “a disease you can die from” (5-point agreement scale) at baseline. Physicians reported on this perception, prognosis and having counseled on this. Staff-assessed comfort with the End-of-Life in Dementia - Comfort Assessment in Dying (EOLD-CAD) scale. Associations between understanding and comfort were assessed with generalized estimating equations, structural equation modeling, and mediator analyses.

**Results:**

A family’s perception of dementia as “a disease you can die from” predicted higher patient comfort during the dying process (adjusted coefficient −0.8, 95% confidence interval (CI): −1.5; -0.06 point increment disagreement). Family and physician combined perceptions (−0.9, CI: −1.5; -0.2; 9-point scale) were also predictive, including in less advanced dementia. Forty-three percent of the families perceived dementia as a disease you can die from (agreed completely, partly); 94% of physicians did. The association between combined perception and higher comfort was mediated by the families’ reporting of a good relationship with the patient and physicians’ perception that good care was provided in the last week.

**Conclusions:**

Awareness of the terminal nature of dementia may improve patient comfort at the end of life. Educating families on the nature of dementia may be an important part of advance care planning.

## Background

The number of people living with dementia will more than triple by 2050 [1], and more family and professional caregivers will provide end-of-life care. We studied if and how caregiver understanding of the progressive and terminal nature of dementia relates to patient comfort when dying.

Understanding the clinical trajectory of dementia may be the basis of high-quality palliative care at the end of life, along with the need “to diagnose dying” [2,3]. Qualitative studies have indicated that families may have little understanding of the natural course of dementia [4,5]. A US study in nursing home patients with advanced dementia showed that if families had limited understanding of the poor prognosis and clinical course of advanced dementia, patients were more likely to undergo burdensome interventions [6]. Retrospective work in diverse settings in patients with terminal diseases linked recognition of dying to fewer diagnostic and therapeutic interventions [7] and to patients more frequently being at peace with their situation [8].

To our knowledge, no prospective work has studied the association between a lack of understanding of the course of dementia and patient outcomes, such as comfort in the dying process, which palliative care specialists find most important in end-of-life decision making [9]. Further, research on end of life in dementia is mostly limited to advanced dementia [6,10-12]. Many patients do not progress to advanced dementia, but die earlier from comorbid disease or dementia-related health problems, so caregiver understanding of dementia may be relevant in earlier stages.

We conducted a nationwide prospective study that included patients in variable stages of dementia and studied family and physician perspectives that influenced end-of-life care. We assessed if family and physician understanding of the progressive and terminal nature of dementia predicts patient comfort while dying, and if this is mediated by care processes around family decision making [5,13], and quality of care provided.

## Methods

Between January 2007 and July 2010, 34 long-term care facilities from each of the 12 provinces in The Netherlands participated in the Dutch End Of Life in Dementia (DEOLD) study. Data were provided from family members and physicians of patients with dementia. The main goals were to describe treatment, care and patient- and family-level outcomes, and factors associations with outcome [11,14]. The 28 nursing homes and 6 residential homes with psychogeriatric units studied were selected for variability in relevant characteristics [14], for example, facility size (ranging from 11 to 210 “psychogeriatric” beds, mostly for dementia), and availability of a palliative care unit. These facilities represented the country average with respect to family’s perceived quality of care as reported in public online databases [14]. We report on 17 physician teams (28 facilities) that collected data on 372 residents with a diagnosis of dementia upon admission to the facility. The homes recruited families and the 58% who participated were not demographically different from non-participants. The study obtained ethics approval from the Medical Ethics Committee of the VU University Medical Center (no 2006/179) and families provided informed consent before taking part.

### Data collection

Facilities recruited family members deemed most involved in the patient’s care up to a year before conclusion of data collection. Families reported their understanding of the nature of dementia eight weeks after the patient’s admission to the facility (baseline), and semi-annually. The eight-week time frame before the baseline assessment allowed for the physician care planning meeting with the family, which is required within six weeks of admission [15]. Physicians were surveyed within two weeks of the patient’s death and families after two months.

### Understanding variables

**Figure 1:**
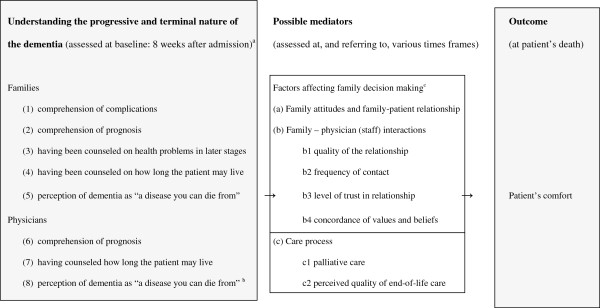
**Framework guiding analyses. **^a^Referring to the assessment at eight weeks after admission, or ((3), (4) and (7)) the period between admission and eight weeks after admission. ^b^Mid-way study, for proximity to family assessment throughout the data collection period, or, for newly employed physicians, soon after being employed. ^c^Possible mediators (a) and (b) are factors related to (more balanced) families’ decision making as described by the theoretical model of Caron *et al.*[5].

Figure 1 (left box) lists the concepts used to define families’ baseline understanding of dementia. As in previous US work [6], comprehension of complications (item 1) was defined as understanding the types of health problems patients may experience in the later stages of dementia. We asked both families and physicians to estimate life expectancy (prognosis; items 2 and 6), with response options “shorter than one month,” “one through six months,” “seven through twelve months,” “longer than twelve months,” and “don’t know.” We dichotomized these categories into a prognosis of 12 months or less versus longer than 12 months and don’t know.

“Having been counseled” referred to receiving information on the type of health problems patients may experience in later stages of dementia (item 3) and on how long the patient may live (item 4). The general perception of whether dementia is a disease you can die from (items 5 and 8) was assessed at baseline for the families and at a year after start of data collection for the physicians, or soon after their employment by the participating home. This perception was rated with five (score 1 to 5) response options: “completely agree,” “partly agree,” “neither agree nor disagree,” “partly disagree” and “completely disagree,” with a “don’t know” option for families only. We considered the respondent to have agreed with the statement if they agreed “completely” or “partly.” For regression analyses, we combined “don’t know” with the middle category of “neither agree, nor disagree,” and we summed families’ and physicians’ 1 to 5 scores resulting in total scores between 2 and 10.

### Possible confounders

Possible confounding factors for which we adjusted were families’ highest completed educational level (combined into a four-level hierarchical variable) and whether the assessor of comfort at death was a physician or a nurse since physicians may report higher levels of comfort [16]. We also adjusted for three variables as they related to the time of the patient’s death: families’ baseline understanding, the physician’s assessment of perception of dementia, and time since the first death in the study, because of trends in treatment and outcome [17,18].

### Possible mediators

Possible mediators of understanding of the progressive nature of dementia and patient outcome were care processes and other family factors identified by Caron *et al.* as related to decision making, such as attitudes, relationships and interaction [5,13] (Figure 1, middle box). Table 1 shows the possible mediators as defined in the DEOLD study.

**Table 1:** Possible mediators and associations between perception of dementia and patient comfort when dying (EOLD-CAD)

**Possible mediators for family and physician perception of dementia as “a disease you can die from” and comfort**	***P*-value GEE regression for adjusted**^**a **^**analyses**	
	**Association with perception of dementia**	**Association with EOLD-CAD comfort score**
**a and b. Factors affecting family decision making** (Caron, Griffith and Arcand, 2005) [5]		
**a. Family attitudes and family-patient relationship**		
Family indicates being critical considering care for resident (3 categories; family, baseline)	0.40	0.03, interaction 0.08
Preference for decision making on care and treatment (family, baseline)		
- family prefers to decide him/herself versus physician, or shared	0.39	0.96, interaction 0.08
- family prefers to leave decisions to the treating physicians, versus self or shared	0.24	0.89, interaction 0.10
Family’s degree of confidence that understood what patient would and would not have wanted with respect to his/her health care and treatment (three categories, family, after death)	0.37	0.17, interaction 0.01
Families’ evaluation of quality of the relationship with patient in terms of intensity and how warm before the patient’s dementia (five categories, family, baseline)^b^ (Mediator)	0.046	0.01, interaction 0.28
**b. Family – physician (or family – health care team including physician) interactions**		
**b1 Quality of the family-physician relationship**		
Physician’s perception on how well family could cope with the patient’s situation, in general (three categories, physician, after-death assessment)	0.62	<0.001, interaction 0.93
Additional person involved in (discussions about) care for the patient in the last month of life (relative who had not or hardly been involved before) (physician, after-death assessment)	0.45	0.06, interaction 0.60
Physician’s satisfaction with how the communication on directives, goals of treatment, and care with the patient’s representative was going (5-point, physician, after-death assessment)	0.97	0.12, interaction 0.008
Family’s satisfaction with how the communication with the physician(s) was going (discussions on future care, goals of treatment, and care in the last phase of life) since previous assessment (zero to six months before) (5-point, family, after death)	0.49	0.65, interaction 0.11
**b2 Frequency of contact with physicians or team**		
Family spoke to elderly care physicians in the last week of the patient’s life (zero to seven days, after-death assessment)	0.24	0.36, interaction 0.39
Family did not spent time in the nursing home in the last month of the patient’s life (family, after-death assessment)	0.94	0.24, interaction 0.02
Patient received visitors in last week of life, according to nurse or physician (four categories, physician, after death)	0.40	0.49, interaction 0.75
**b3 Level of trust in relationship with physicians**		
Family indicated trust in physicians (5-point, family, after-death)	0.01	0.69, interaction 0.95
Family had relationship of trust with physician, as perceived by physician (5-point, physician, after-death)	0.57	0.48, interaction 0.39
**b4 Concordance of values and beliefs between family and physicians or team**		
Degree to which all persons involved in treatment(s) and care (nursing home staff and family members), agreed about the best treatment(s) and care in the last month of the patient’s life as perceived by family (three categories, family, after death)	0.67	0.08, interaction 0.55
Degree to which all persons involved in treatment(s) and care, agreed about the best treatment(s) and care in the last month of the patient’s life as perceived by physicians (three categories, physicians, after death)	0.27	0.22, interaction 0.01
**c. Care process**		
**c1 Palliative care (indicators)**		
Treatment goal that took priority: palliative (including symptomatic)^c^ versus other goal (physician)		
- at day of death	0.75	0.54, interaction 0.45
- at baseline assessment	0.42	0.22, interaction 0.69
Any burdensome interventions in the last week of life [6]	0.88	0.71, interaction 0.81
**c2 Perceived quality of end-of-life care**		
Family’s overall rating of care that patient received in the last week of life (5-point, family after death)	0.15	0.86, interaction 0.76
Physician’s overall rating of (quality of) care that patient received in the last week of life (5-point, physician, after death)^b^ (Mediator)	0.02	0.005, interaction 0.56

EOLD-CAD, End-of-Life in Dementia-Comfort Assessment in Dying score; GEE, generalized estimating equations.

^a^Adjusted for potential confounders: assessment of EOLD-CAD by physician versus nurse (last column only), family education, and three variables that related to the time of the patient’s death: families’ baseline understanding, the physician’s assessment of perception of dementia mid-way data collection, and time since the first death in the study. *P*-values represent models without imputation (n = 122 to 143). Significance did not differ for models with simple imputation (n = 160).

^b^Values of the two mediators: quality of the relationship: excellent 46%, good 41%, moderate 10%, fair 3%, poor 1%. Physician’s overall rating of quality of care: excellent 6%, very good 37%, good 57%, fair 1%, poor 1%. When combining the last three categories to improve the distribution when used as an outcome variable in the association with the perception of the dementia, the *P*-value for quality of the relationship was 0.02, and for physician’s overall rating of the quality of care: 0.051.

^c^Palliative and symptomatic treatment goals both refer to comfort, quality of life and well-being, but differ as to whether prolongation of life is desirable.

Regarding care processes as possible mediators, we used indicators for palliative care and overall assessments of quality of care to limit confounding of single treatment by patient condition (for example, antibiotic treatment may reduce discomfort in pneumonia [19]). Similar to previous US work [6], we defined potentially burdensome interventions as hospitalization, emergency room visit, or new or ongoing parenteral therapy or tube feeding.

### Outcome

The assessment of a patient’s comfort during the dying process used End-of-Life in Dementia–Comfort Assessment in Dying (EOLD-CAD; staff assessment, Figure 1, right box) [20], a validated 14-item scale which assesses quality of dying [21] and has better psychometric properties and user friendliness than other such measures [16]. Total scores range from 14 to 42 with higher scores representing more comfort.

### Subgroups by dementia severity

Dementia severity was assessed with the highly discriminative Bedford Alzheimer Nursing Severity-Scale (BANS-S) [22,23]. To compare with US work [6], we also defined advanced dementia as a Cognitive Performance Scale (CPS) [24] score of 5 or 6 and a Global Deterioration Scale (GDS) [25] score of 7.

### Analyses

Power calculations with α = 0.05, R^2^ = 0.17, 80% power, ICC = 0.05 and a mean of 3 patients per cluster (physician), indicated that 135 patients sufficed. We performed generalized estimating equations (GEE) regression analyses to adjust for clustering of patients with physicians, with EOLD-CAD-scores as the dependent variable, and understanding as the independent variable. Confidence intervals (95% CI) were calculated. As demonstrated to be appropriate in previous work [26], missing items, if maximum 4 of 12, were imputed with patient means to calculate a total score. Later missing data on physician’s perceptions of dementia (11%) was mostly due to staff turnover and was imputed in the combined physician-family score by the mean of the not very variable physician’s score. Analyses were performed without imputation, and, to check for possible differences, also with simple imputation.

We performed mediator analyses according to the MacArthur approach [27]. In brief, we examined the possible mediators assessing in adjusted GEE analyses, first, if there was a significant (*P* <0.05) association between the understanding variable and the possible mediator, and, second, if there also was an association or interaction between the possible mediator and outcome in presence of the understanding variable. We used probit, ordinal probit, Poisson or linear regression as appropriate for the type of possible mediator being the outcome in the first series of analyses. If associations were significant in both steps (Table 1), the mediator was tested in structural equation modeling (SEM) to model multiple associations and quantify direct and indirect effects using M-plus version 6.11 (2011) (Muthen & Muthen, Los Angeles, CA, USA). Other analyses were performed with PASW 18.0.0 (2009) (PASW Statistics for Windows, Version 18.0, SPSS Inc., Chicago, IL, USA).

To examine possible family-physician communication and resulting consensus, we additionally tested correlations between families’ and physicians’ understanding using Pearson’s correlation coefficients. We calculated Cohen’s kappa and 95% CI for agreement between family and physicians responding to the same dichotomous understanding items. Agreement was slight for kappa <0.2; fair, 0.2 to 0.4; moderate, 0.4 to 0.6; substantial 0.6 to 0.8; almost perfect >0.8 [28].

## Results

**Figure 2:**
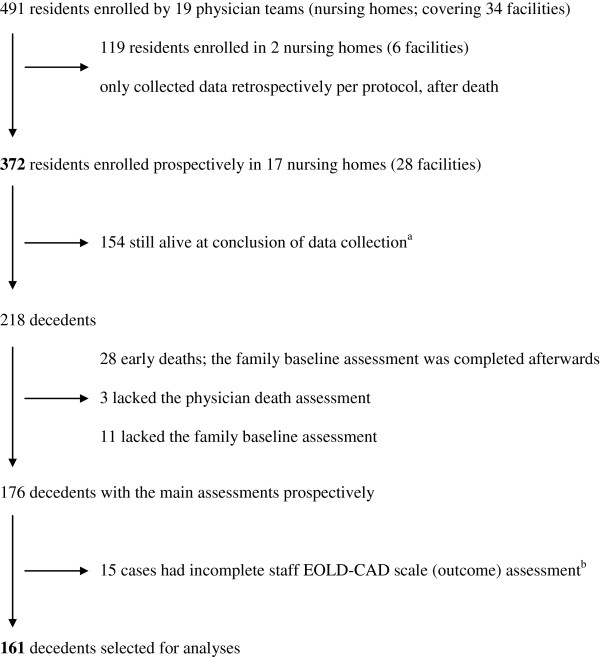
**Selection of patients for analyses.** EOLD-CAD, End-of-Life in Dementia-Comfort Assessment in Dying. ^a^Vital status 1 July 2010; until then, after-death assessments were being performed. Survival status was monitored until summer 2011. ^b^Reasons for incomplete assessment included that staff was not present when the patient was dying (unexpected death, found dead, died in hospital) and delay in completing the death assessment (for example, due to staff change or death immediately after admission), in case we no longer required a death assessment.

Of the 372 patients, 218 (59%) died within the data collection period (Figure 2). We selected 161 patients with a complete EOLD-CAD assessment and a prospective baseline family assessment of understanding the progressive nature of dementia.

Most (69%) decedents were female; mean age at death was 86.0 (SD 6.3; Table 2). Most family (64%) and physicians (61%) were also female. The physicians (73) had a mean of 11.5 years (SD 8.1) of clinical experience in nursing homes. Patients’ mean length of stay until death was 1.0 year (SD 0.7; range 0.1 to 3.1 years). The mean EOLD-CAD-score for comfort (staff assessment) was 34.1 (SD 5.6).

**Table 2:** **Characteristics of patients (n = 161), their families and physicians, outcome and potential confounders**^**a**^

**Patients**	
Female gender,%	69
Age at death (mean, SD)	86.0 (6.3)
Length of stay in nursing home (mean number of years, SD)	1.0 (0.7)
Dementia severity (mean BANS-S score, SD)	
- at baseline	14.3 (4.2)
- at last semi-annual assessment before death^b^	15.7 (4.2)
Advanced dementia (GDS 7 and (CPS 5 or 6)),%	
- at baseline	13
- one month before death	41
**Families**	
Female gender,%	64
Age at baseline assessment (mean, SD)	60.3 (11.7)
Relationship to deceased resident,%	
- child (including child and legal representative or other combinations)	59
- spouse	19
- other	22
Highest completed education,%^c^	
- none or primary/elementary school	6
- (high school preparing for) technical/trade school	56
- high school preparing for BSc or MSc	10
- BSc or MSc degree	28
**Physicians** (weighted for number of patients they treated)	
Female gender,%	61
Age at assessment of perception of dementia	43.1 (8.6)
Experience as a physician in nursing home (mean number of years, SD)	11.5 (8.1)
Full time equivalent (mean, SD)	0.78 (0.17)
**Outcome and potential confounders**^c^	
EOLD-CAD score for comfort (mean, SD)^d^	34.1 (5.6)
Assessment of EOLD-CAD by physician (versus nurse under supervision of physician),%	47
Time between patient’s death, mean number of years (SD), and	0.80 (0.67)
- baseline assessment (family, and most physician understanding variables)	
- physician’s assessment of perception of dementia	0.18 (0.74)
- death of the first subject in study	1.93 (0.72)

BANS-S, Bedford Alzheimer Nursing Severity-Scale (possible range 7 to 28; scores of 17 and higher represent severe dementia [29]); CPS, Cognitive Performance Scale; EOLD-CAD, End-of-life in Dementia-Comfort Assessment in Dying scale (possible range: 14 to 42, higher scores represent better comfort); GDS, Global Deterioration Scale; SD, standard deviation.

^a^Missing values were 3 for both BANS-S assessments, 11 for advanced dementia at baseline, 1 for relationship family to deceased resident, 2 for family education, 8 for physician demographics, 17 for physician experience, 21 for full time equivalent, 8 for assessment of EOLD-CAD by physician, 8 for time between death and physician’s assessment of perception of dementia, and there were no missing values for the other characteristics.

^b^The last assessment before death was the baseline assessment in 50% of cases, and a semi-annual assessment (the first through the fifth) in the other 50% of cases.

^c^Highest completed education was also a potential confounder.

^d^Nurses’ mean EOLD-CAD scores were not significantly lower than physician’s mean ratings of different patients in the DEOLD study (33.8 SD 6.7 versus 34.4 SD 4.4; *P* = 0.53; no pairwise comparison possible; adjusted for in analyses even though it did not change results).

Less than half (41%) died with advanced dementia. Causes of death listed on the death certificate did not differ significantly between those with advanced and less advanced dementia regarding, for example, cachexia (23% versus 18%; *P* = 0.44), infection (35% versus 36%; *P* = 0.93), dehydration (41% versus 39%, *P* = 0.80) or cardiovascular disease (44% versus 49%; *P* = 0.54), nor did it differ regarding dementia as a cause (85% versus 87%; *P* = 0.75), as an immediate cause (38% versus 27%; *P* = 0.15) or a contributing cause (47% versus 60%; *P* = 0.12) of death.

### Understanding

Half of families reported understanding of the complications that can occur in dementia (Table 3). Only 15% of families expected death within 12 months. A minority reported having been counseled on these issues. Whereas family perceptions of dementia “as a disease you can die from” varied (43% agreed completely or partly), almost all (94%) physicians agreed. In 39% of cases both parties agreed. Over a quarter of families (28%) did not know.

**Table 3:** Variables referring to the understanding of the dementia and associations with patient’s comfort when dying

**Variable**	**Response% or mean (SD)**	**Mean EOLD-CAD patient comfort score at the end of life (SD)**	**Difference in mean patient comfort relative to the reference group or per unit increase on scale**	
			**Unadjusted (95% CI)**	**Adjusted**^**a **^**(95% CI)**
**Families** (baseline assessment, upon admission)				
(1) Comprehension of complications		(n = 161; overall: 34.1 SD 5.6)	(n = 161)	(n = 151)
- understood	50	33.9 (6.5)	reference	reference
- not understood	32	33.8 (4.7)	−0.1 (−2.1; 1.9)	−0.3 (−2.4; 1.9)
- refused (do not know and similar comments)	18	35.3 (4.5)	1.4 (−0.7; 3.4)	0.9 (−1.1; 2.9)
			*P* = 0.31^b^	*P* = 0.50^b^
(2) Comprehension of prognosis: life expectancy		(n = 161; overall: 34.1 SD 5.6)	(n = 161)	(n = 151)
- 12 months or less (<1 month: 1%, 1 to 6 months: 5%,				
7 to 12 months: 9%)	15	34.1 (7.5)	reference	reference
- more than 12 months	32	33.7 (5.7)	−0.4 (−3.5; 2.7)	−0.6 (−3.6;2.3)
- do not know	53	34.4 (5.0)	0.3 (−2.9; 3.4)	−0.6 (−3.5;2.4)
			*P* = 0.82^b^	*P* = 0.91^b^
(3) Having been counseled on health problems in later stages		(n = 161; overall: 34.1 SD 5.6)	(n = 161)	(n = 151)
-yes	39	34.7 (5.7)	reference	reference
-no	61	33.8 (5.6)	−0.9 (−2.6; 0.9)	−0.9 (−2.8;1.1)
(4) Having been counseled on how long patient may live		(n = 160; overall:	(n = 160)	(n = 150)
		34.1 (5.6)		
-yes	21	34.5 (6.2)	reference	reference
-no	79	34.0 (5.5)	−0.6 (−2.7; 1.6)	−0.6 (−2.8;1.6)
(5) Perception of dementia as “a disease you can die from”		(n = 160 overall)	(n = 160)	(n = 150)
- 1 to 5 scale, coefficient b^c^	2.5 (1.2)	34.2 SD 5.6	b = −*0.7 (−1.5; -0.01)*	b = −*0.8 (−1.5;-0.06)*
**-** completely agree	29	35.1 (5.6)	reference	reference
**-** partly agree	14	34.9 (7.1)	−0.1 (−3.4; 3.1)	−0.1 (−3.3; 3.1)
**-** neither agree, nor disagree	13	34.8 (4.6)	−0.3 (−2.7; 2.0)	−1.0 (−3.6; 1.6)
**-** partly disagree	8	33.8 (4.4)	−1.2 (−4.3; 1.8)	−1.6 (−4.3;1.1)
**-** completely disagree	9	31.5 (5.5)	*−3.6 (−6.5; -0.7)*	*−3.6 (−6.5;-0.7)*
**-** do not know	28	33.5 (5.6)	−1.5 (−4.5; 1.4)	−1.5 (−4.5; 1.6)
**Physicians**				
(6) Comprehension of prognosis: perceived life expectancy (baseline)		(n = 150; overall: 34.4 SD 5.4)	(n = 150)	(n = 138)
- 12 months or less (<1 month: 1%, 1 to 6 months: 9%, 7 to 12 months: 16%)	25	33.6 (6.5)	reference	reference
- more than 12 months	59	34.4 (5.0)	0.8 (−1.5; 3.2)	0.3 (−2.1; 2.8)
- do not know	16	35.5 (5.1)	1.9 (−0.9;4.7)	1.2 (−1.7; 4.1)
			*P* = 0.39^b^	*P* = 0.67^b^
(7) Having counseled how long the patient may live (baseline)		(n = 150; overall:	(n = 150)	(n = 138)
		34.4 SD 5.4)		
-yes	21	34.9 (4.9)	reference	reference
-no	79	34.2 (5.5)	−0.7 (−2.7; 1.3)	−0.8 (−2.8;1.3)
(8) Perception of dementia as “a disease you can die from” (midway study)		(n = 144 overall)	(n = 144)	(n = 138)
- 1 to 5 scale, coefficient b^c^	4.7 (0.8)	34.1 SD 5.7	b = −1.0 (−2.4; 0.4)	b = −1.0 (−2.2; 0.2)
**-** completely agree	85	34.2 (5.9)	reference	reference
**-** partly agree	9	35.8 (2.3)	*1.5 (0.1; 2.9)*	*1.8 (0.5; 3.1)*
**-** neither agree, nor disagree	3	30.5 (5.4)	−3.7 (−7.7; 0.3)	−3.1 (−7.3;1.2)
**-** partly disagree	0	-	-	-
**-** completely disagree	3	29.6 (7.3)	−4.6 (−11; 2.2)	−4.9 (−11;1.3)
**Families and physicians**				
Perception of dementia as “a disease you can die from,” 2 to 10 scale, coefficient b^c^	8.2 (1.5)	(n = 160 overall) 34.2 SD 5.6	(n = 160) b = −*0.8 (−1.4;-0.2)*	(n = 143) b = −*0.9 (−1.5;-0.2)*^d^

EOLD-CAD, End-of-life in Dementia-Comfort Assessment in Dying scale (possible range: 14 to 42, higher scores represent better comfort).

^a^Adjusted for potential confounders: assessment of EOLD-CAD by physician versus nurse; for three variables as they related to the time of the patient’s death: families’ baseline understanding, the physician’s assessment of perception of dementia, and time since the first death in the study; and family education when applicable (for example, no adjustment for family variables in analyzing associations with physician variables only). Adjustment was without imputation which explains the lower n. With simple imputation of mean or median as appropriate, confidence intervals were minimally smaller and coefficients were similar.

^b^The *P*-values refer to GEE versions of ANOVA (unadjusted *P*-value) or ANCOVA (adjusted).

^c^b is the regression coefficient for 1-point increment disagreement, where “neither agree, nor disagree” is combined with “don’t know” (families).

^d^The coefficients and confidence intervals were similar (adjusted: b = −*0.9 (−1.5; -0.3))* when eight cases in which the physician completed the after-death assessment and baseline assessment at the same time, were excluded from the analyses.

There were multiple significant intercorrelations between families’ understanding variables, for example, between comprehension of complications and the four other variables. Physician’s prognosis correlated with having counseled families on this. Families’ and physicians’ agreement on prognosis and counseling was fair (kappa 0.25; 95% CI, 0.07 to 0.42; kappa 0.22; CI, 0.03 to 0.40, respectively; not in Table). Families’ and physicians’ perceptions of dementia did not correlate (r = 0.02, *P* = 0.78). Families’ perception of dementia in the last semi-annual assessment before death (available for 84 cases) did not differ from their perception at baseline (mean 2.6 SD 1.3 versus 2.5 SD 1.4, *P* = 0.52 for pairwise comparison) and the assessments over time correlated significantly (r = 0.49; *P* <0.001; not in Table).

### Understanding and outcome

Families’ understanding of complications, prognosis and having been counseled on these, was unrelated to patient’s comfort when dying (Table 3) as was physicians’ prognosis and having counseled on this at admission. However, families’ perception of dementia as a disease you can die from was associated with higher patient’s comfort in a stepwise fashion for higher agreement (adjusted −0.8 point less comfort; increment more disagreement; CI, -1.5; -0.06). The unadjusted EOLD-CAD means for “completely disagree” versus “completely agree” were 31.5 SD 5.5, versus 35.1 SD 5.6 (difference −3.6, effect size: 0.6), and the adjusted difference was also −3.6 EOLD-CAD points (Table 3). For physicians, there was no significant association but, qualitatively, mean comfort when treated by the few physicians who disagreed was considerably lower. There was no stepwise decrease for those agreeing completely versus partly; however, contrasting agreement (completely and partly) versus no agreement, there was a significant association with comfort (unadjusted difference −4.4; CI, -8.6; -0.1; not in Table).

### Combined understanding, outcome and mediation

For subsequent analyses, we combined families’ and physicians’ agreement into the 2 to 10 scale for perception of dementia (Table 3, lowest row shows a significant association, adjusted and unadjusted analyses were similar) reflecting relevance of both perceptions to outcome in examining possible mediators including those referring to family-physician interaction (GEE analyses; Table 1). Advanced dementia did not, but dementia severity as measured by the last BANS-S assessment somewhat affected the association between perception of dementia and higher comfort (b = −0.6, CI −1.2; 0.02). However, the association did not differ by dementia severity (*P* = 0.11 for interaction).

**Figure 3:**
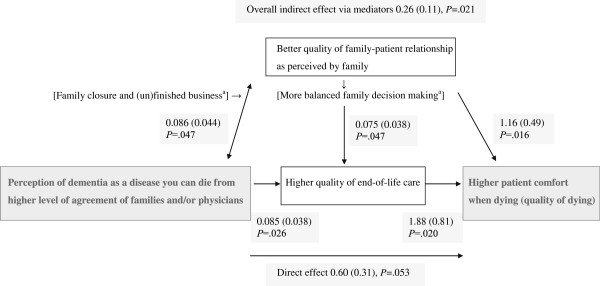
**Model of understanding the progressive nature of dementia and associations with outcome and mediators.** Coefficients, standard errors (between brackets), and *P*-values for the associations in the SEM model are shown. GEE, generalized estimating equations; SEM, structural equation modeling. ^a^Factors between brackets were not measured in our study, but refer to interpretations of a better quality of family-patient relationship as perceived by family as a mediator.

Table 1 shows that we examined two to four items of each of categories a-c in Figure 1. A reportedly positive relationship (warm and intense) between patient and family before the dementia was a significant mediator regarding patient’s comfort while dying and families’ and physicians’ perception of dementia, as was physician report that good quality of care was provided in the week prior to death (Table 1). Figure 3 shows the results of SEM analyses. The two mediators remained significant in the inclusive, final model, and were significantly correlated. The overall indirect effect was significant, although the indirect effect of the separate mediators was not (*P* = 0.11 and 0.08, respectively; not in Figure), as was the direct effect (*P* = 0.053). The overall indirect effect of the two mediators explained over a quarter (0.26/0.86) of the association between perception of the dementia and patient’s comfort.

## Discussion

A family’s understanding of dementia as “a disease you can die from” assessed shortly after a patient’s admission independently predicted higher comfort in Dutch nursing home patients dying with dementia, including those with less advanced dementia. To our knowledge, this is the first prospective study that suggests that an early basic understanding of dementia as a terminal disease may be prerequisite to providing comfort at the end of life. Even though some die with, rather than directly from, the dementia, such understanding seems rational because dementia shortens life, and death is difficult to predict [11,30]. Further, many patients never progress to advanced dementia, but we found similar causes of death for less advanced dementia.

The difference in adjusted EOLD-CAD-means of “completely disagree” versus “completely agree” of nearly 4 (−3.6) points (effect size 0.6), could imply that the dying family member had observed, for example, pain and shortness of breath “a lot” versus “not at all.” Only about half (43%) of families agreed that dementia is a disease you can die from, whereas 94% of physicians did. There were indications that patients were less comfortable when treated by physicians who disagreed. We examined mechanisms whereby the combined perceptions of families and physicians, driven mostly by the larger variability in the families’ perception, translated into higher patient comfort and found that this was mediated by higher quality of end-of-life care as perceived by physicians and a better family-patient relationship reported by the family, the two of which were associated as well. Closer relationships and secure attachment styles have been associated with less increase of problem behavior over time [31,32]. Further, Dutch work in cancer care found that patient’s awareness of dying was associated with acceptance of dying [8]. Possibly acceptance of dying is more difficult with a poor family-patient relationship due to unfinished business and lack of closure.

We found no differences by stage of dementia. The other understanding items we examined, namely comprehension of complications, prognosis and counseling, were unrelated to patient comfort. Compared to these items, the broad perception of dementia as a disease you can die from may be more relevant to palliative care and decision making and less confounded by patient condition or socially desirable responding.

Because consensus on prognosis and planning of palliative care is important to physicians [3,4,33], families’ perception of dementia as a terminal disease may help physicians in providing better end-of-life care. Families’ perceptions were important even though family views are less influential in decision making in The Netherlands than in the US due to a culture of Dutch physicians being more directive [34]. In The Netherlands, physicians are based in nursing homes and work frequently with dying patients and curative interventions are frequently withheld in dementia [12,35]. In our study, almost all physicians perceived dementia as a disease you can die from. This may be different in other countries, where curative interventions are commonly provided [11]. Cross-national work may be important; for example, when explored, in our Dutch data we could not replicate findings of Mitchell *et al.*[6] of family perceptions being related to potentially burdensome treatment at the end of life.

### Limitations and strengths

Although associations should be interpreted as causal with caution, our prospective study with patients enrollment upon admission to a nursing home was based on a previous, thorough, conceptualization of decision making [5,13], and we employed established methods for mediator analyses in the final step.

The perception of the dementia was measured ahead of patient’s comfort, but some mediators were measured in parallel. Multiple in-between assessments would have been preferable. The quality of the family-patient relationship referred to the relationship before the patient developed dementia so does not fulfill criteria for a mediator requiring temporal precedence [27]. Although we did not measure the quality of the present relationship nor the families’ psychological conditions in detail, we feel this mediator is a proxy for current relationship and relates to decision making. We are supported in this by our finding of trends of family decision making in the months before death mediating the association between perception and patient’s comfort, but power was insufficient to estimate coefficients because such decisions were made in only about half of the cases. Further, we could not identify specific treatments as mediators and physician’s perception of quality of end-of-life care was a subjective assessment. The power to detect associations between life expectancy and comfort was limited due to few families providing any prognosis. Further, exploring possible selection bias, we found that family comfort assessments were not different with missing staff outcome assessment (18/179 cases), and associations with family comfort as an outcome with the combined perception of dementia as a disease you can die from did not differ either. However, families were more likely to disagree with dementia as a disease you can die from when staff comfort assessment was missing, which implies we even underestimated families’ disagreement with the perception.

Average length of stay (follow-up) until death was short (one year) for which we adjusted our analyses and follow-up until death was as long as three years. Finally, we were able to explain a considerable part of the main association with two broad evaluations as mediators, but not all. The association of comfort with the broad perception of dementia may reflect a complex interplay involving multiple factors.

## Conclusion and implications for practice and research

We found that caregivers’ understanding of the progressive and terminal nature of dementia at the time of a patient’s admission to a long-term care facility predicts patient comfort when dying. Such understanding may be fundamental to the provision of high-quality palliative care at the end of life.

The difference in comfort between patients whose caregivers understand that dementia is a terminal disease and those who do not raises the concern that patients have suffered unnecessarily during the dying process. Informing families that dementia is “a disease you can die from,” even in less advanced stages, may be part of advance care planning. Families themselves might also benefit if a decline in a patient’s health status triggers conversations about dying [3], including strategies for acceptance. A minority of physicians in our study had counseled families despite the fact that many families did not understand the poor prognosis in dementia. Family education strategies suitable for all levels of education [36] should be tested as to whether they increase understanding of the natural course of dementia, and whether this then influences a patient’s comfort when dying. Further, effects of the acknowledging of a disease as terminal may be examined in other chronic, progressive diseases, such as chronic heart failure and COPD.

This work was presented at the annual meeting of the Gerontological Society of America (GSA), Boston, 19 November 2011.

## Abbreviations

BANS-S: Bedford Alzheimer Nursing Severity-Scale; CI: Confidence interval; CPS: Cognitive Performance Scale; DEOLD: Dutch End Of Life in Dementia; EOLD-CAD: End-of-Life in Dementia - Comfort Assessment in Dying; GDS: Global Deterioration Scale; GEE: generalized estimating equations; SEM: structural equation modeling.

## Competing interests

The authors declare that they have no competing interests.

## Authors’ contributions

JTvdS and DLK had full access to all of the data in the study and take responsibility for the integrity of the data and the accuracy of the data analysis. JTS and MWR contributed to conception and design and obtained funding. JTS acquired the data and drafted the manuscript. JTS, BDP, DLK, MWR and LD analyzed and interpreted the data and critically revised the manuscript for important intellectual content. JTS and DLK did the statistical analysis. JTS, BDP and MWR carried out administrative, technical or material support. JTS, BDP, MWR and LD supervised the study. All authors read and approved the final manuscript.

## Author’s information

Jenny T. van der Steen was also employed by the Department of Public and Occupational Health of the EMGO Institute during data collection for the study.

## Pre-publication history

The pre-publication history for this paper can be accessed here:

http://www.biomedcentral.com/1741-7015/11/105/prepub

## References

[B1] World Health Organization Dementia: A Public Health Priority 2012 Geneva, Switzerland: WHO

[B2] Ellershaw J Ward C Care of the dying patient: the last hours or days of life BMJ 2003 326 30 34 10.1136/bmj.326.7379.30 12511460 PMC1124925

[B3] Bern-Klug M Calling the question of "possible dying" among nursing home residents: triggers, barriers, and facilitators J Soc Work End Life Palliat Care 2006 2 61 85 10.1300/J457v02n03_06 17387090

[B4] Gessert CE Forbes S Bern-Klug M Planning end-of-life care for patients with dementia: roles of families and health professionals Omega (Westport) 2000 42 273 291 12569923 10.2190/2mt2-5gyu-gxvv-95ne

[B5] Caron C Griffith J Arcand M Decision making at the end of life in dementia: how family caregivers perceive their interactions with health care providers in long-term- care settings J Appl Gerontol 2005 24 231 247 10.1177/0733464805275766

[B6] Mitchell SL Teno JM Kiely DK Shaffer ML Jones RN Prigerson HG Volicer L Givens JL Hamel MB The clinical course of advanced dementia N Engl J Med 2009 361 1529 1538 10.1056/NEJMoa0902234 19828530 PMC2778850

[B7] Veerbeek L Van Zuylen L Swart SJ Jongeneel G van der Maas PJ van der Heide A Does recognition of the dying phase have an effect on the use of medical interventions? J Palliat Care 2008 24 94 99 18681245

[B8] Lokker ME van Zuylen L Veerbeek L van der Rijt CC van der Heide A Awareness of dying: it needs words Support Care Cancer 2012 20 1227 1233 10.1007/s00520-011-1208-7 21688164 PMC3342506

[B9] Raijmakers NJ van Zuylen L Costantini M Caraceni A Clark JB De Simone G Lundquist G Voltz R Ellershaw JE Heide AV Issues and needs in end-of-life decision making: an international modified Delphi study Palliat Med 2012 26 947 953 10.1177/0269216311423794 21969309

[B10] Sachs GA Dying from dementia N Engl J Med 2009 361 1595 1596 10.1056/NEJMe0905988 19828537

[B11] van der Steen JT Dying with dementia: what we know after more than a decade of research J Alzheimers Dis 2010 22 37 55 20847433 10.3233/JAD-2010-100744

[B12] Hughes JC Promoting palliative care in dementia Lancet Neurol 2010 9 25 27 10.1016/S1474-4422(09)70328-X 20083030

[B13] Caron C Arcand M Griffith J Creating a partnership with families in decision making for end-of-life care in Alzheimer disease: the perspective of family caregivers Dementia 2005 4 113 136 10.1177/1471301205049193

[B14] van der Steen JT Ribbe MW Deliens L Gutschow G Onwuteaka-Philipsen BD **Retrospective and prospective data collection compared in the Dutch End of Life in Dementia (DEOLD) study** Alzheimer Dis Assoc Disord 2013 In press 10.1097/WAD.0b013e318293b380 23632265

[B15] Staatsblad 2009 no 131. Besluit zorgplanbespreking AWBZ-zorg [Exceptional Medical Expenses Act, AWBZ] 2012 https://zoek.officielebekendmakingen.nl/stb-2009-131.html

[B16] van Soest-Poortvliet MC van der Steen JT Zimmerman S Cohen LW Klapwijk MS Bezemer M Achterberg WP Knol DL Ribbe MW de Vet HC Psychometric properties of instruments to measure the quality of end-of-life care and dying for long-term care residents with dementia Qual Life Res 2012 21 671 684 10.1007/s11136-011-9978-4 21814875 PMC3323818

[B17] Cohen LW van der Steen JT Reed D Hodgkinson JC van Soest-Poortvliet MC Sloane PD Zimmerman S Family perceptions of end-of-life care for long-term care residents with dementia: differences between the United States and the Netherlands J Am Geriatr Soc 2012 60 316 322 10.1111/j.1532-5415.2011.03816.x 22288500

[B18] van der Steen JT Meuleman-Peperkamp I Ribbe MW Trends in treatment of pneumonia among Dutch nursing home patients with dementia J Palliat Med 2009 12 789 795 10.1089/jpm.2009.0049 19622013

[B19] van der Steen JT Pasman HR Ribbe MW Van Der Wal G Onwuteaka-Philipsen BD Discomfort in dementia patients dying from pneumonia and its relief by antibiotics Scand J Infect Dis 2009 41 143 151 10.1080/00365540802616726 19065450

[B20] Volicer L Hurley AC Blasi ZV Scales for evaluation of End-of-Life Care in Dementia Alzheimer Dis Assoc Disord 2001 15 194 200 10.1097/00002093-200110000-00005 11723370

[B21] van Soest-Poortvliet MC van der Steen JT Zimmerman S Cohen LW Munn J Achterberg WP Ribbe MW de Vet HC Measuring the quality of dying and quality of care when dying in long-term care settings: a qualitative content analysis of available instruments J Pain Symptom Manage 2011 42 852 863 10.1016/j.jpainsymman.2011.02.018 21620642

[B22] Volicer L Hurley AC Lathi DC Kowall NW Measurement of severity in advanced Alzheimer's disease J Gerontol 1994 49 M223 M226 10.1093/geronj/49.5.M223 8056941

[B23] Bellelli G Frisoni GB Bianchetti A Trabucchi M The Bedford Alzheimer Nursing Severity scale for the severely demented: validation study Alzheimer Dis Assoc Disord 1997 11 71 77 9194953 10.1097/00002093-199706000-00003

[B24] Morris JN Fries BE Mehr DR Hawes C Phillips C Mor V Lipsitz LA MDS Cognitive Performance Scale J Gerontol 1994 49 M174 M182 10.1093/geronj/49.4.M174 8014392

[B25] Reisberg B Ferris SH de Leon MJ Crook T The Global Deterioration Scale for assessment of primary degenerative dementia Am J Psychiatry 1982 139 1136 1139 7114305 10.1176/ajp.139.9.1136

[B26] van der Steen JT Gijsberts MJ Knol DL Deliens L Muller MT Ratings of symptoms and comfort in dementia patients at the end of life: comparison of nurses and families Palliat Med 2009 23 317 324 10.1177/0269216309103124 19346275

[B27] Kraemer HC Kiernan M Essex M Kupfer DJ How and why criteria defining moderators and mediators differ between the Baron & Kenny and MacArthur approaches Health Psychol 2008 27 2 Suppl S101 S108 18377151 10.1037/0278-6133.27.2(Suppl.).S101 PMC3376898

[B28] Landis JR Koch GG The measurement of observer agreement for categorical data Biometrics 1977 33 159 174 10.2307/2529310 843571

[B29] van der Steen JT Volicer L Gerritsen DL Kruse RL Ribbe MW Mehr DR Defining severe dementia with the Minimum Data Set Int J Geriatr Psychiatry 2006 21 1099 1106 10.1002/gps.1618 16955417

[B30] van der Steen JT Heymans MW Steyerberg EW Kruse RL Mehr DR The difficulty of predicting mortality in nursing home residents Eur Geriatr Med 2011 2 79 81 10.1016/j.eurger.2011.01.005

[B31] Fauth E Hess K Piercy K Norton M Corcoran C Rabins P Lyketsos C Tschanz J Caregivers' relationship closeness with the person with dementia predicts both positive and negative outcomes for caregivers' physical health and psychological well-being Aging Ment Health 2012 16 699 711 10.1080/13607863.2012.678482 22548375 PMC3430821

[B32] Perren S Schmid R Herrmann S Wettstein A The impact of attachment on dementia-related problem behavior and spousal caregivers' well-being Attach Hum Dev 2007 9 163 178 10.1080/14616730701349630 17508315

[B33] Bern-Klug M Gessert CE Crenner CW Buenaver M Skirchak D Getting everyone on the same page": nursing home physicians' perspectives on end-of-life care J Palliat Med 2004 7 533 544 10.1089/jpm.2004.7.533 15353097

[B34] Helton MR van der Steen JT Daaleman TP Gamble GR Ribbe MW A cross-cultural study of physician treatment decisions for demented nursing home patients who develop pneumonia Ann Fam Med 2006 4 221 227 10.1370/afm.536 16735523 PMC1479435

[B35] van der Steen JT Kruse RL Ooms ME Ribbe MW van der Wal G Heintz LL Mehr DR Treatment of nursing home residents with dementia and lower respiratory tract infection in the United States and The Netherlands: an ocean apart J Am Geriatr Soc 2004 52 691 699 10.1111/j.1532-5415.2004.52204.x 15086647

[B36] Volandes AE Paasche-Orlow MK Barry MJ Gillick MR Minaker KL Chang Y Cook EF Abbo ED El-Jawahri A Mitchell SL Video decision support tool for advance care planning in dementia: randomised controlled trial BMJ 2009 338 b2159 10.1136/bmj.b2159 19477893 PMC2688013

